# Polymorphism analysis of *pfmdr1* gene in *Plasmodium falciparum* isolates 11 years post-adoption of artemisinin-based combination therapy in Saudi Arabia

**DOI:** 10.1038/s41598-021-04450-x

**Published:** 2022-01-11

**Authors:** Hesham M. Al-Mekhlafi, Aymen M. Madkhali, Ahmed A. Abdulhaq, Wahib M. Atroosh, Ahmad Hassn Ghzwani, Khalid Ammash Zain, Khalid Y. Ghailan, Hassan A. Hamali, Abdullah A. Mobarki, Talal H. Alharazi, Zaki M. Eisa, Yee-Ling Lau

**Affiliations:** 1 Medical Research Centre, Jazan University, Jazan, Kingdom of Saudi Arabia; 2 Vector-Borne Diseases Research Group, Jazan University, Jazan, Kingdom of Saudi Arabia; 3 Department of Parasitology, Faculty of Medicine and Health Sciences, Sana’a University, Sana’a, Yemen; 4 Department of Medical Laboratory Technology, Faculty of Applied Medical Sciences, Jazan University, Jazan, Kingdom of Saudi Arabia; 5 Department of Parasitology, Faculty of Medicine, University of Malaya, 50603 Kuala Lumpur, Malaysia; 6 Department of Microbiology and Parasitology, Faculty of Medicine and Health Sciences, University of Aden, Aden, Yemen; 7 Faculty of Public Health and Tropical Medicine, Jazan University, Jazan, Kingdom of Saudi Arabia; 8 Department of Clinical Laboratory Sciences, College of Applied Medical Sciences, University of Hail, Hail, Kingdom of Saudi Arabia; 9 Department of Microbiology and Immunology, Faculty of Medicine and Health Sciences, Taiz University, Taiz, Yemen; 10 Saudi Centre for Disease Prevention and Control, Ministry of Health, Jazan, Kingdom of Saudi Arabia

**Keywords:** Malaria, Parasite genetics

## Abstract

A total of 227 *Plasmodium falciparum* isolates from Jazan region, southwestern Saudi Arabia were amplified for the *P. falciparum multi-drug resistance 1* (*pfmdr1*) gene to detect point mutations 11 years after the introduction of artemisinin-based combination therapy (ACT) in Saudi Arabia. The *pfmdr1* 86**Y** mutation was found in 11.5% (26/227) of the isolates while the N86 wild allele was detected in 88.5%. Moreover, 184**F** point mutations dominated (86.3%) the instances of *pfmdr1* polymorphism while no mutation was observed at codons 1034, 1042 and 1246. Three *pfmdr1* haplotypes were identified, N**F**SND (74.9%), NYSND (13.7%) and **YF**SND (11.4%). Associations of the prevalence of 86**Y** mutation and **YF**SND haplotype with participants’ nationality, residency and parasitaemia level were found to be significant (*P* < 0.05). The findings revealed significant decline in the prevalence of the *pfmdr1* 86**Y** mutation in *P. falciparum* isolates from Jazan region over a decade after the implementation of ACT treatment. Moreover, the high prevalence of the N**F**SND haplotype might be indicative of the potential emergence of CQ-sensitive but artemether-lumefantrine-resistant *P. falciparum* strains since the adoption of ACT. Therefore, continuous monitoring of the molecular markers of antimalarial drug resistance in Jazan region is highly recommended.

## Introduction

Malaria is a leading cause of morbidity and death worldwide, particularly in developing countries in the tropical and subtropical regions. This vector-borne disease is caused by five species of the genus *Plasmodium* namely *Plasmodium falciparum*, *P. vivax*, *P. malariae*, *P. ovale* and *P. knowlesi*, and is transmitted to humans by the bite of infected female *Anopheles* mosquitoes. *Plasmodium falciparum* is the most virulent species, causing the most severe cases of malaria and more than 90% of malaria mortality globally^1^. Currently, about half of the world’s population is at risk of malaria, with approximately 229 million cases and about 400,000 malaria deaths recorded worldwide in 2019^2^. About 95% of all global malaria cases and deaths have occurred in Africa, especially in sub-Saharan Africa countries^3^. Moreover, it has been estimated that about 3% or ~ 6.4 million cases of malaria in 2019 were caused by *Plasmodium vivax*^4^.

Following its discovery during World War II, chloroquine (CQ) became the official first-line treatment for malaria for decades. However, as mentioned above, cases of CQ-resistant malaria emerged in the late 1950s and then spread worldwide^5,6^. Consequently, in some African countries, sulfadoxine-pyrimethamine (SP) was used as a new first-line treatment for malaria. However, SP-resistant strains emerged abruptly and have now become widespread throughout Africa^7,8^. In 2005, the WHO recommended using artemisinin-based combination therapies (ACT) as first- and second-line treatments for uncomplicated *P. falciparum* malaria^9^. Unfortunately, since then, studies have reported that delayed parasite clearance and ACT treatment failure due to artemisinin resistance is widespread in the Greater Mekong Subregion of Southeast Asia^10,11^.

The *P. falciparum multi-drug resistance 1* (*pfmdr1*) gene (also known as *P-glycoprotein homologue 1*; *Pgh1*), which is located on chromosome 5, has been associated with parasite susceptibility to a variety of currently available antimalarial drugs including chloroquine (CQ), lumefantrine (LMF), amodiaquine (AQ), mefloquine (MFQ), quinine (QN) and artemisinin (AS)^12,13^. Several *pfmdr1* single-nucleotide polymorphisms (SNPs) have been described to date, among which the five most common are N86Y, Y184F, S1034C, N1042D and D1246Y^14^. Previous studies have concluded that, for these five SNPS, substitution of asparagine by tyrosine at codon 86 (N86Y), which is the most prevalent mutation in Asian and African parasites, enhances parasite resistance to CQ and AQ but at the same time increases susceptibility to a wide range of first-line antimalarial drugs including LMF, MFQ and dihydroartemisinin (DHA)^13,15^. Interestingly, previous studies have also reported a significant decline in the 86**Y** point mutation a few years following the adoption of ACT^16,17^. Therefore, surveillance of changes in the prevalence of *pfmdr1* SNPs might provide early warning of the emergence of resistance to ACT.

In Saudi Arabia, a national malaria control programme was established in 1948 and since then, substantial progress has been made in reducing the presence of malaria in the country^18^. Saudi Arabia is now in the elimination phase, and malaria transmission is restricted to specific foci in the southwestern parts of the country, particularly in the regions of Jazan and Aseer^19,20^. However, while the number of annual malaria cases decreased steadily between 2000 and 2014, an increasing trend has been observed since 2015, with 3658 cases reported in 2020, and 82.6% of those cases were in Jazan region^20,21^. In 2007, CQ was replaced by ACT for the treatment of uncomplicated falciparum malaria, with artesunate + sulfadoxine-pyrimethamine or mefloquine (AS + SP or MFQ) as the first-line and artemether-lumefantrine (AL) as the second-line treatment^22,23^. Resistance to CQ in Saudi Arabia was first identified in 1992 based on the increased incidence of clinical and parasitological treatment failure^24^. Subsequently, prevalence rates of up to 100% of the *P. falciparum chloroquine resistance transporter* (*pfcrt*) 76 T point mutation (the primary driver of CQ resistance) were reported^25,26^.

However, data on the *pfmdr1* point mutation genotypes and haplotypes circulating in Saudi Arabia are lacking. Therefore, this study aimed to determine the frequency and distribution of *pfmdr1* point mutations and related haplotypes in *P. falciparum* isolates from Jazan region 11 years post-adoption of ACT. Such information is essential to guide health policy-makers on the selection and the monitoring of the country’s ACT treatment policy.

## Results

**Figure 1:**
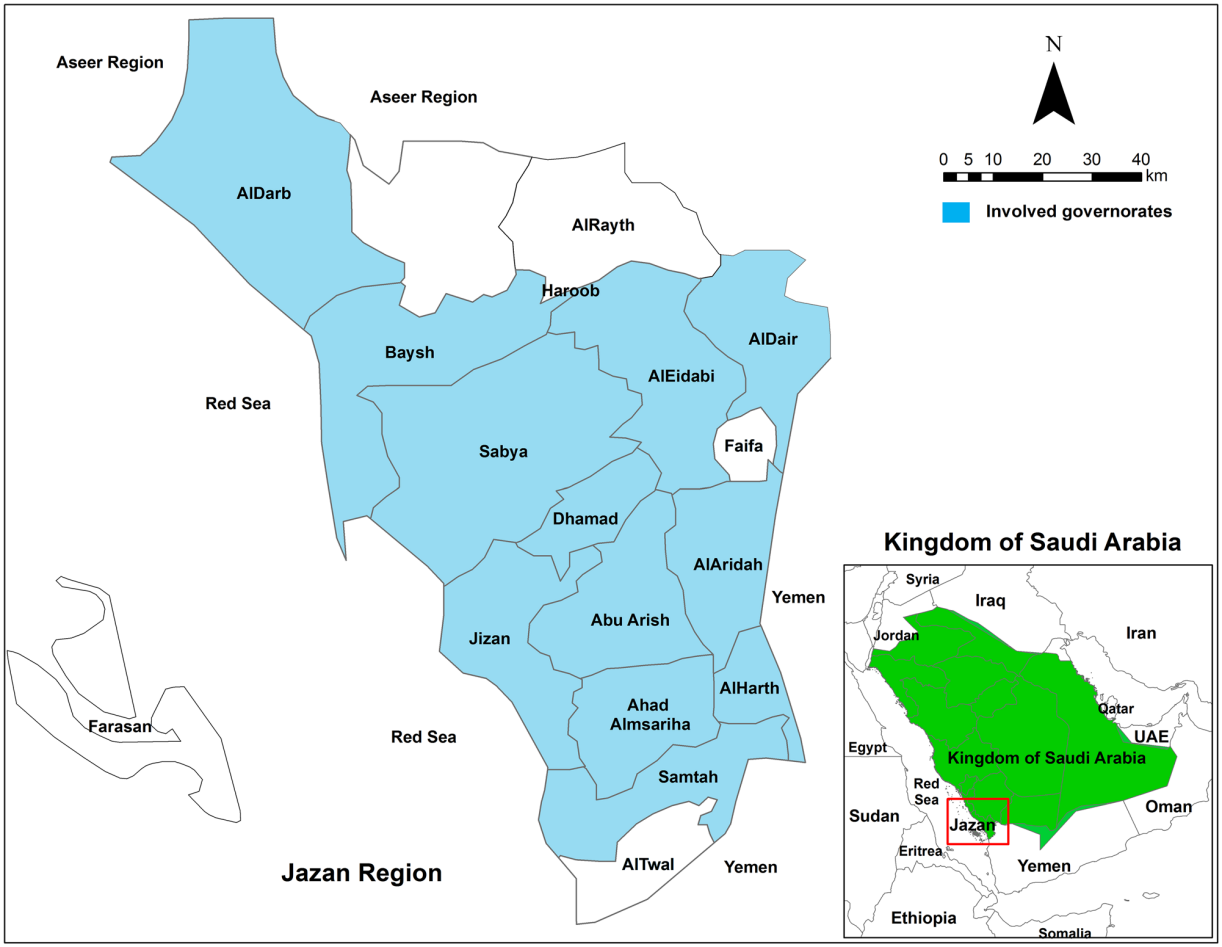
A map of the study area in Jazan region, southwestern Saudi Arabia (12 governorates were involved in this study). The map was originally generated using ArcGIS software version 10.8.1.

Two hundred and twenty-seven falciparum malaria-positive patients (82.1% males and 17.2% females) were involved in this study (Table 1). The patients were aged between 2 and 78 years and the mean age was 30.7 ± 11.6 years. The majority of the participants (69.6%) were from rural areas and 19.4%, 13.7% and 11.9% of the participants were from Baysh, Jazan and Alharth governorates, respectively. Malaria isolates were collected from 12 of the 17 governorates of Jazan region (Fig. 1). Approximately 66% (150/227) of the participants were non-Saudi while about 34% were Saudi. The majority of the non-Saudi patients were from Yemen (46%) followed by Pakistan (18.7%) and India (12%). With regard to parasitaemia (asexual parasite density), 55.1% (125/227) of the patients had moderate-to-high parasitaemia while 44.9% had low parasitaemia (Table 1).

**Table 1:** General characteristics of malaria patients participated in the study (n = 227).

Variables	Number	%
**Age groups**		
< 30	106	46.7
≥ 30	121	53.3
**Gender**		
Male	188	82.8
Female	39	17.2
Residence		
Rural	158	69.6
Urban	69	30.4
**Nationality**		
Saudi Arabia	77	33.9
Yemen	69	30.4
Pakistan	28	12.3
India	18	7.9
Sudan	11	4.8
Egypt	8	3.5
Bangladesh	8	3.5
Ethiopia	5	2.2
Philippine	2	0.9
Syria	2	0.4
**Parasitaemia**		
Low	102	44.9
Moderate	70	30.9
High	55	24.2

### Frequency and distribution of *pfmdr1* mutations and haplotypes

Out of the 250 isolates collected, the *pfmdr1* gene was successfully amplified in 227 isolates (90.8%), with 86.3% (196/227) of the isolates showing at least one point mutation at one codon (Table 2). Of the five codons screened, three codons of *pfmdr1* (1034, 1042 and 1246) were found to consist of 100% wild alleles. On the other hand, mutated alleles at codons 86**Y** and 184**F** were found in 11.5% and 86.3% of the studied isolates, respectively. Only three *pfmdr1* haplotypes were found to be circulating in the study area. The most dominant haplotype (74.9%) was the N**F**SND single-mutated haplotype, which represents the substitution of phenylalanine amino acid at position 184. Also, the **YF**SND double-mutated haplotype was found in 26 isolates (11.4%), which was carrying the mutated amino acids tyrosine for codon 86 and phenylalanine for codon 184. On the other hand, the wild haplotype, NYSND, was found in 13.7% of the isolates.

**Table 2:** Frequency and number of *pfmdr1* point mutations and related haplotypes for *P. falciparum* isolates from Jazan, Saudi Arabia (n = 227).

Marker	Type of mutations	Number	%
**Codon**			
*Pfmdr1*-86	Wild (N86)	201	88.5
	Mutated (86**Y**)	26	11.5
*Pfmdr1*-184	Wild (Y184)	31	13.7
	Mutated (184**F**)	196	86.3
*Pfmdr1*-1034	Wild (S1034)	227	100
	Mutated (1034**C**)	0	0
*Pfmdr1*-1042	Wild (N1042)	227	100
	Mutated (1042**D**)	0	0
*Pfmdr1*-1046	Wild (D1046)	227	100
	Mutated (1046**Y**)	0	0
**Haplotype**			
NYSND	Wild	31	13.7
N**F**SND	Single	170	74.9
**YF**SND	Double	26	11.4

Mutant alleles are bold and underlined.

### Associations of *pfmdr1* mutations with demographic factors

Some associations were found between the *pfmdr1* point mutations and haplotypes and the patients’ demographic variables. The results demonstrated that the highest prevalence of the 86**Y** point mutation was found in the isolates from Yemeni patients (20.3%) followed by those from Sudanese patients (18.2%). Accordingly, significantly higher percentages of isolates carrying the *pfmdr1* 86**Y** mutated allele and **YF**SND double-mutated haplotype were found in isolates from non-Saudi patients when compared with isolates from Saudi patients (14.7% *vs.* 5.2%; χ^2^ = 4.501; *P* = 0.034). Moreover, significantly higher percentages of the 86**Y** point mutation and **YF**SND haplotype were observed in isolates from participants living in urban areas than those from patients living in rural areas (20.3% vs. 7.6%; χ^2^ = 7.632; *P* = 0.006). On the other hand, significant associations were not found between the *pfmdr1* mutations and haplotypes and the participants’ age group and gender (*P* > 0.05) (Table 3).

**Table 3:** Associations of *pfmdr1* mutant alleles and related haplotypes detected in *P. falciparum* isolates from Jazan region with patients’ demographic factors (n = 227).

Marker	Age group			Gender			Nationality			Residency		
	< 30	≥ 30	*P*	Females	Males	*P*	Saudi	Non-Saudi	*P*	Rural	Urban	*P*
*Pfmdr1*-86**Y**	11 (10.4)	15 (12.4)	0.634	5 (12.8)	21 (11.2)	0.783^†^	4 (5.2)	22 (14.7)	0.034*	12 (7.6)	14 (20.3)	0.006*
*Pfmdr1*-184**F**	92 (86.8)	104 (86.0)	0.854	36 (92.3)	160 (85.1)	0.233	67 (87.0)	129 (86.0)	0.833	136 (86.1)	60 (87.0)	0.859
NYSND	14 (13.2)	17 (14.0)	0.854	3 (7.7)	28 (14.9)	0.233	10 (13.0)	21 (14.0)	0.833	22 (13.9)	9 (13.0)	0.859
N**F**SND	81 (76.4)	89 (73.6)	0.620	31 (79.5)	139 (73.9)	0.467	63 (81.8)	107 (71.3)	0.085	124 (78.5)	46 (66.7)	0.059
**YF**SND	11 (10.4)	15 (12.4)	0.634	5 (12.8)	21 (11.2)	0.783^†^	4 (5.2)	22 (14.7)	0.034*	12 (7.6)	14 (20.3)	0.006*

Mutant alleles are bold and underlined.

All values are number (%).

*Pfmdr1*-1034, *Pfmdr1*-1042 and *Pfmdr1*-1046 were of wild type and thus, not included in the analysis.

*Significant association (*P* < 0.05).

^†^The difference was examined using Fisher’s exact test (otherwise, Chi Square test was used).

**Figure 2:**
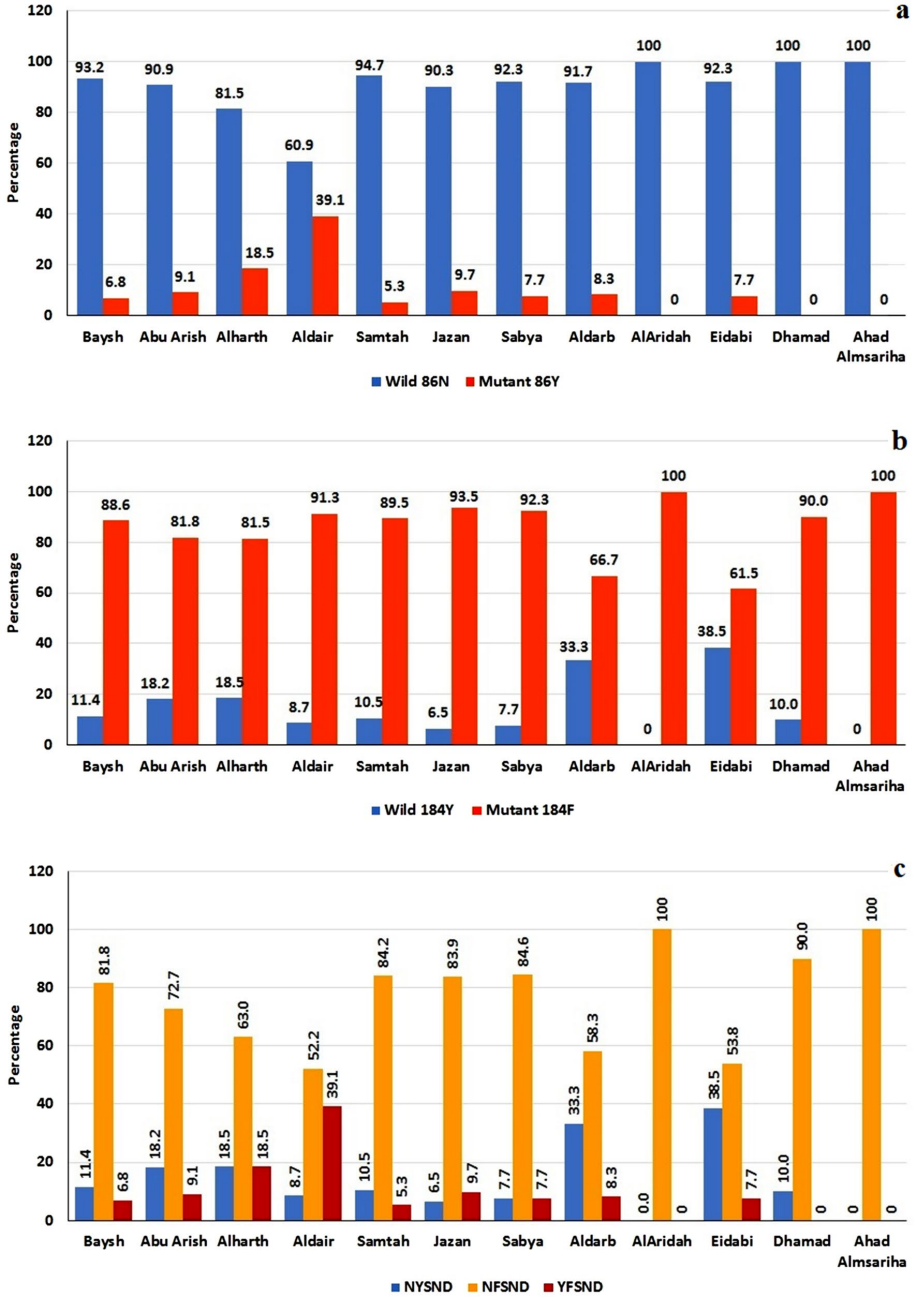
Distribution of *pfmdr1* N86Y and Y184F mutations and haplotypes according to governorates involved in the study in Jazan region. (**a**) *pfmdr1* N86Y. (**b**) *pfmdr1* Y184F. (c) *pfmdr1* haplotypes.

In addition, the results showed that mutations in the *pfmdr1* gene varied significantly among the studied governorates (Fig. 2). It was found that the percentages of the *pfmdr1* 86**Y** mutation and the **YF**SND haplotype were the highest in isolates collected from Aldair governorate (39.1%; 9/23) followed by those from Alharth governorate (18.5%; 5/27) while these markers were not reported in Alaridah, Dhamad and Ahad Almsariha governorates (χ^2^ = 24.021; *P* = 0.013). The opposite situation was observed for the distribution of the N**F**SND single-mutated haplotype in those governorates: N**F**SND was detected in all isolates from Alaridah, Dhamad and Ahad Almsariha governorates while the lowest percentages were in isolates from Aldair (52.2%) and Eidabi (53.8%) governorates (χ^2^ = 22.776; *P* = 0.019). The distribution of the *pfmdr1* 184**F** point mutation among the studied governorates was not statistically significant (*P* > 0.05).

### Association of *pfmdr1* mutations with parasitaemia

**Figure 3:**
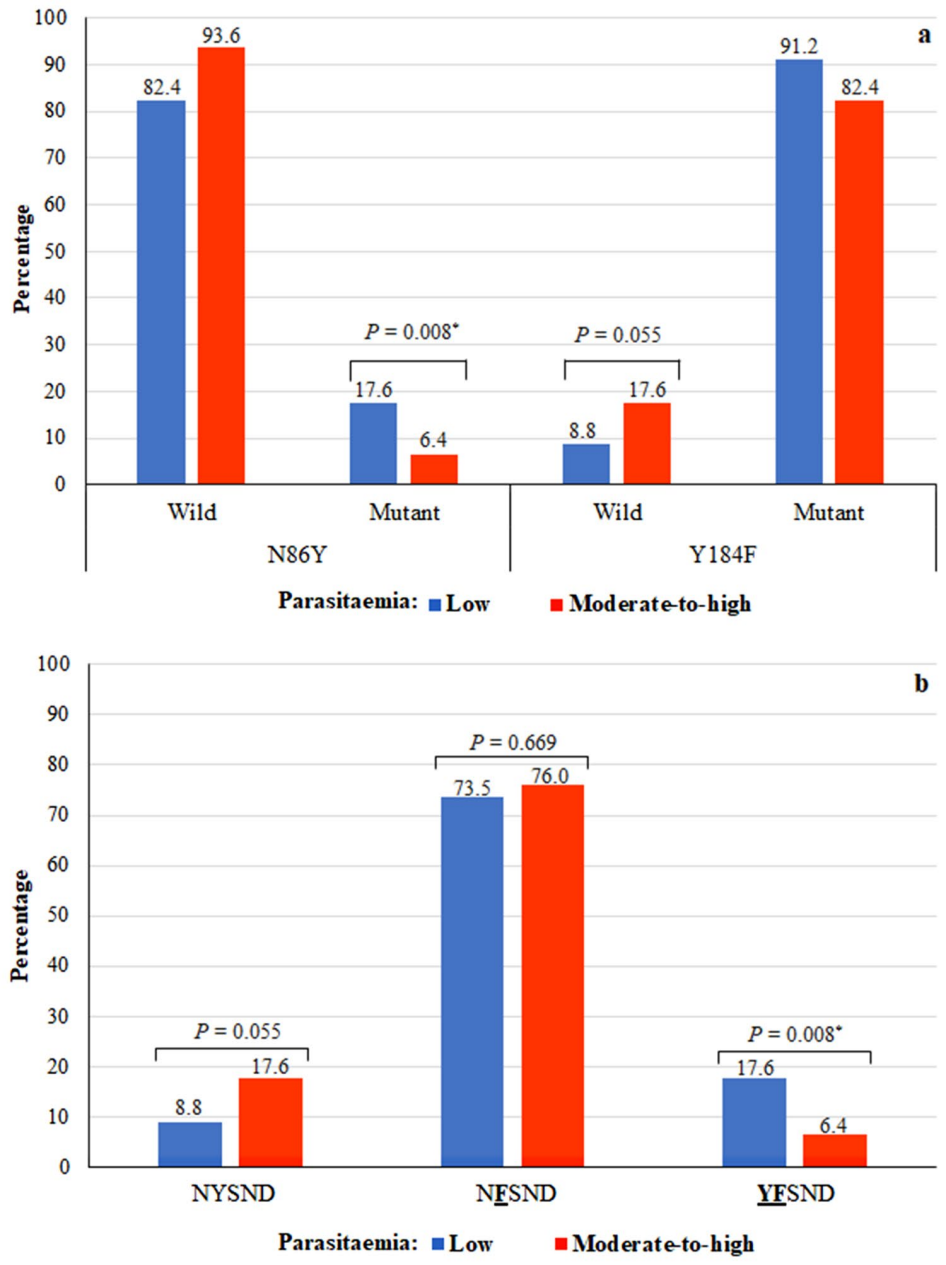
Association of *pfmdr1* N86Y and Y184F mutations and haplotypes with parasitaemia in isolates from Jazan region. Parasitaemia levels: low (< 1000 parasites/μl of blood); moderate-to-high (≥ 1000 parasites/μl of blood). Mutant alleles are bold and underlined. *Significant association (*P* < 0.05). (**a**) *pfmdr1* N86Y and Y184F mutations. (**b**) *pfmdr1* haplotypes.

The association of *pfmdr1* mutations and haplotypes with parasitaemia level was also examined (Fig. 3). The percentages of the mutated 86Y allele and the **YF**SND haplotype in isolates from participants with a low parasitaemia level were significantly higher than in the isolates of those participants with moderate-to-high parasitaemia (17.6% *vs.* 6.4%; χ^2^ = 7.006; *P* = 0.008). Likewise, the percentage of the *pfmdr1* 184**F** mutation was higher in isolates from patients with a low parasitaemia level (91.2%) as compared to those with moderate-to-high parasitaemia (82.4%); however, the difference was not statistically significant (*P* = 0.055). Similarly, significant associations were not found between the percentages of the NYSND and the N**F**SND haplotypes and parasitaemia (*P* > 0.05).

## Discussion

The current study provides information on genetic polymorphisms in the *pfmdr1* gene in *P. falciparum* isolates from Jazan region, a low malaria transmission area, 11 years after the discontinuation of CQ and the adoption of ACT in its place. The findings revealed that 11.5% and 86.3% of the isolates carried the *pfmdr1* 86**Y** and 184**F** point mutations, respectively. Accordingly, three *pfmdr1* haplotypes were identified, with the single (N**F**SND) and double (**YF**SND) mutated haplotypes found in 74.9% and 11.4% of the isolates, respectively.

The prevalence of the *pfmdr1* 86**Y** mutation found by the current study is far lower than that reported by the only two studies available from Saudi Arabia, which were conducted in Jazan region in 2010 and 2012^26,27^. In 2010, the 86**Y** mutation was found in 40.4% of 109 *P. falciparum* isolates from the Aseer and Jazan regions^27^. Two years later, 30% of 165 *P. falciparum* isolates from Jazan region were found to be carrying the *pfmdr1* 86**Y** point mutation^26^. Moreover, the current finding that the 184**F** point mutation dominated the *pfmdr1* polymorphism of the analysed isolates is consistent with the sole local study that reported 96% (159/165) of this mutation in the region despite an approximately 10% decline demonstrated by our result^26^. Likewise, the current study found no mutations at codons 1034, 1042 and 1246, similar to the study conducted in Jazan region in 2012^26^.

The current findings suggest that there has been a significant decline in the prevalence of *pfmdr1* 86**Y** (and conversely an increase in the wild N86 allele) since the implementation of the ACT treatment policy in Saudi Arabia, which is consistent with previous reports elsewhere^15,17,28,29^. However, a conclusion based on two previous studies^26,27^ and the current study might not be reliable. Therefore, further analyses of the SNPs of the *pfmdr1* gene over time using archived samples are required to enable a better understanding of the trend in *pfmdr1* mutations in Jazan region. Interestingly, the current findings corroborate the results of our separately published study on the same isolates, which found a significant decline in the prevalence of *pfcrt* 76T and the re-emergence of *pfcrt* K76 CQ-sensitive *P. falciparum* strains in Jazan region^30^. Altogether, about two-thirds (69.2%; 18/26) of the isolates that carried the *pfmdr1* 86Y mutation were also found to be carrying the *pfcrt* 76 T mutation. The *pfmdr1* 86Y point mutation is the second most important molecular marker of CQ resistance after *pfcrt* 76 T, and several studies have suggested that there may be a synergistic association between these *pfcrt* and *pfmdr1* point mutations^31,32^. Moreover, a remarkable decline in the prevalence of mutant *pfcrt* 76** T** and *pfmdr1* 86**Y** alleles has also been observed in low malaria transmission settings where drug pressure is likely to be lower^33^.

In Yemen, a neighbouring country with high malaria transmission, previous studies showed that the prevalence of the 86**Y** mutation was higher than that reported by the current study, varying between 16 and 20% in the governorates of Hadhramoud (southeastern Yemen) and Hodeidah (northwestern Yemen)^34–36^. However, striking results were reported in Taiz governorate (southwestern Yemen) in which all of the 50 isolates analysed were found to be carrying the wild N86 allele^37^. On the other hand, mutation at codon 184 was found at a fixation level (100%)^34,36^. Interestingly, a high prevalence of mutations (up to 70%) for 1034**C** and 1042**D** has been reported across Yemen^34,36^. In Iran, the 86**Y** mutation was detected in 23% of isolates while mutations at the other four codons were not detected in any isolate^38^. Variations in the geographical distribution of *pfmdr1* mutations at codons 86 and 184 and haplotypes have also been reported in different African and Asian countries^13^. The current study also found that there was significant variation in the distribution of *pfmdr1* polymorphism, with the highest prevalence of the 86**Y** mutation and **YF**SND haplotype in isolates from Aldair and Alharth governorates. These governorates border Yemen and most of the isolates were from Yemeni patients. Thus, the higher percentage of 86**Y** and **YF**SND might be attributed to the source of malaria infection circulating in northern Yemen.

The current study also demonstrated that three *pfmdr1* haplotypes are circulating in Jazan region, with the single-mutant N**F**SND (74.9%) dominant followed by the wild NYSND (13.7%), and the double-mutant **YF**SND (11.4%) haplotypes. This pattern is different from that reported in 2012, where 66% and 31% of the examined isolates carried the single N**F**SN and double **YF**SN haplotypes while only 3% carried the wild NYSN haplotype (codon 1246 was not involved)^26^. Studies from other countries such as Yemen and Ghana have reported up to five different haplotypes^32,36^.

Thus, the mutations of *pfmdr1* gene are complex. Moreover, the mechanism for modulating multiple antimalarial drug resistance in *P. falciparum* parasites as well as their associations with the mutations of other genes such as *pfcrt* is not well understood. Mutation at codon 86 has been associated with CQ resistance in several studies from different regions while mutations at other codons of the *pfmdr1* gene have been found to have complementary roles^13^. Some previous studies have reported that mutation at codon 86, either alone or in combination with mutations at codons 184 and 1246 (**YF**SN**Y** haplotype), enhances the susceptibility of *P. falciparum* to ACT, specifically AS + MFQ (the first-line treatment in Saudi Arabia)^16,39^. On the other hand, the wild-type *pfmdr1* N86 has been linked to the less sensitive LMF and MFQ phenotypes^40^. Moreover, it has been reported that the carrying of the wild-type N86 together with the mutant 184**F** and wild D1246 (N**F**SND haplotype) enhances parasite tolerance to AL^41,42^. In addition, a selection of the *pfmdr1* 86**Y** and 1246**Y** mutations has been observed post-AQ exposure^43^, whereas mutations at codons 1034**C**, 1042**D** and 1246**Y** have been found to be associated with the low parasite susceptibility to QN^44^. In the current study, these mutations (1034**C**, 1042**D** and 1246**Y**) were not detected. Thus, it could be concluded that QN treatment, which is the third-line treatment (or the rescue treatment) for both uncomplicated and severe falciparum malaria in Saudi Arabia remains effective. However, it should be noted that AQ has not been used in Saudi Arabia. As a whole, the different patterns of circulating *pfmdr1* haplotypes that have been identified thus far should be considered when formulating and updating antimalaria drug strategies in endemic areas.

Interestingly, significant selection of the *pfmdr1* N86 and *pfcrt* K76 alleles after AL treatment has been reported by previous in vivo and in vitro studies^45–47^. Moreover, previous studies have demonstrated that increases in *pfmdr1* copy number, which occurred only with N86 and not with the 86**Y** allele, resulted in a greater risk of failure following treatment with AL or MFQ, either as monotherapy or AS-MFQ combination therapy^48–51^. In the same vein, it was found that N**F**SND haplotype significantly decreased parasite susceptibility to LMF, MFQ and AS when compared with the NYSND wild haplotype, and this suggests a specific role of the *pfmdr1* 184**F** allele in conferring resistance to those antimalarials in *P. falciparum* parasites carrying the multiple *pfmdr1* copy numbers^48,51,52^. In addition, haplotype-specific *pfmdr1*-mediated drug transport has been demonstrated by previous studies, with N**F**SND haplotype showed significantly higher MFQ and LMF transport capacity that resulted in decreased susceptibility to these drugs compared to other haplotypes including NYSND and **Y**YSN**Y**^13,49,51,53^.

The finding of the current study that N**F**SND was the predominant pfmdr1 haplotype, combined with our previous observation of the re-emergence of *pfcrt* K76 allele in Jazan region^30^, suggests a risk for selection and spread of multicopy *pfmdr1* and the emergence of AS-MFQ and AL tolerance or resistance in Saudi Arabia after the ongoing deployment of AS-MFQ and AL. This possibility would have important public health implications for malaria treatment in the country as both MFQ and LMF are important partner drugs of the first- and second-line ACT in Saudi Arabia. Therefore, further studies and continuous surveillance are warranted to closely monitor the changes in the *pfmdr1* copy number, which has been proved as the most sensitive indicator of selection of *P. falciparum* parasites populations by AL and AS-MFQ.

In addition, the current study found significant associations between the *pfmdr1* 86**Y** mutation and the **YF**SND haplotype and the nationality and the residence of the participants while associations with age and gender were not significant. The percentages of 86**Y** and **YF**SND in isolates from non-Saudi patients were significantly higher than in isolates from Saudi patients. This is consistent with the fact that more than half (53.8%; 14/26) of these markers (86**Y** and **YF**SND) were detected in isolates from Yemeni patients. These results corroborate the results of our separately published study on the same isolates that showed that 95% of the isolates from Yemeni patients carried the *pfcrt* 76** T** mutations^30^. In a previous study in Jazan, associations between *pfmdr1* markers and the patients’ nationality and gender were found to be not significant^26^. Similarly, studies from Yemen, Sudan and Qatar found no association of *pfmdr1* mutations with the age and gender of patients^31,35,36,54^. In contrast, a study in Kenya found that males are more likely to harbour CQ-resistant *P. falciparum* parasites with high *pfmdr1* mutations as compared females. This difference was attributed to a practice among males of delayed treatment seeking, which may have placed them at higher risk of accumulating super-infections and developing resistance^29^.

The current study also demonstrated that the percentages of the *pfmdr1* 86**Y** mutation and the **YF**SND haplotype in isolates from patients living in urban areas were almost three times higher than in isolates from patients in rural areas while the percentages of the 184**F** mutation and the N**F**SND haplotype were comparable between both groups. While there is as yet no clear explanation for this finding, a previous study in Burkina Faso attributed a higher rate of CQ failure in urban settings to higher drug pressure in those areas as compared to rural areas^55^. However, conversely, other studies have found a higher prevalence of the 86**Y** mutation in rural areas, and this has been explained by the practice of self-medication^41,56^. In addition, the findings of the current study also revealed that the *pfmdr1* 86**Y** mutation and the **YF**SND haplotype occurred more at low parasitaemia, which is consistent with findings by earlier studies elsewhere^57–59^. Although causality cannot be inferred, the association between the *pfmdr1* 86**Y** mutation and the parasitaemia level has been linked to multiplicity of infection and parasite virulence, with parasites with decreased virulence carrying the mutant *pfmdr1* 86**Y** allele^59,60^.

Some limitations of the current study should be acknowledged. First, causal relationships between pfmdr1 markers and the factors studied could not be confirmed due to the cross-sectional design of the study. Second, the reported molecular findings were not linked with malaria treatment outcome among the study participants. Third, a novel genotype, 86**F**, which occurs as a result of asparagine amino acid substitution by phenylalanine at *pfmdr1* codon 86 (N86F) instead of the common substitution by tyrosine (N86Y), has been reported in few *P. falciparum* isolates from different countries including Swaziland^61^, Sudan^62^, Afghanistan^63^, and the Philippines^64^. The Afl-III enzyme used by the current study in genotyping *pfmdr1* codon 86 was found unable to differentiate between both 86**Y** and 86**F** mutant alleles^65^; thus, in our study all digestions with this enzyme were recorded as 86**Y** isolates.

## Conclusions

The current study revealed a significant decline in the prevalence of the *pfmdr1* 86**Y** mutation in *P. falciparum* isolates from Jazan region after over a decade has passed since the introduction of ACT treatment in Saudi Arabia. The results showed a high prevalence of the wild-type alleles N86 and D1246 and of the mutant-type allele 184**F.** The results also showed that the N**F**SND haplotype was the predominant haplotype. These findings may be indicative of increased susceptibility to CQ and decreased sensitivity to AL (second-line treatment) as well as to MFQ (an alternative of the SP partner drug in AS-SP treatment). These observations have crucial implications for the selection and monitoring of ACT in the region and for the identification of approaches that will provide protection against the development of resistance. Moreover, further evaluation and continued monitoring of molecular markers of antimalarial drug resistance in the region is warranted to inform treatment policies and maintain the effectiveness of the malaria elimination programme.

## Methods

### Ethics statement

Ethical approved for this study was obtained from the Ethics Committee of Jazan University (Ref. No. REC39/6-261). Another approval was also obtained by the Health Affairs Directorate of King Fahd Central Hospital, Jazan, Saudi Arabia (Registry No. 086). Before data and sample collection, the patients, or their guardians in the case of children, were informed about the aims and procedures of the study. Then, written signed informed consent was obtained from all adult participant's and from parents/guardians of participants below 18 years. The patients were treated for malaria at the respective hospitals according to the national malaria drug policy. All experiments were performed in accordance with relevant guidelines, regulations and good laboratory practice.

### Study area

This cross-sectional study was carried out between April and December 2018 in the Jazan region, which located in the southwestern part of Saudi Arabia (16° 17′ North, 42° 43′ East). Febrile patients who were suspected to have malaria and who presented at the selected healthcare facilities were involved in this study.

Jazan region is bordered by the Red Sea to the west and by Yemen to the south. The region covers a total area of 11,671 km^2^ and has a population of approximately 1.4 million^66^. At the administrative level, Jazan region is divided into 17 governorates, including Jazan, the capital city (Fig. 1). Jazan region comprises three major landforms: 1) coastal lowlands at an altitude of < 400 m above sea level with aggregated rainfall of < 100 mm/year, 2) foothill areas at an elevation of 400–600 m with aggregated rainfall of 100–200 mm/year, and 3) mountainous areas (highlands) at an elevation of > 2000 m with aggregated rainfall of > 300 mm/year^67^. The region also has a few valleys and streams as well as 15 dams for the conservation of drinking and irrigation water^68^.

Although malaria transmission has been interrupted throughout the country, a few foci for malaria transmission still persist in the regions of Jazan and Aseer. The transmission level peaks between December and March; however, malaria cases are reported throughout the year^20^. Different *Anopheles* species have been identified as vectors for malaria in Jazan region, including *An. arabiensis*, the principal vector, *An. sergentii*, known as the desert malaria vector and *An. Dthali*^69^. In general, malaria in Jazan region is mainly imported by migrant workers, particularly legal and illegal emigrants from Yemen^19^. Nonetheless, dozens of autochthonous malaria cases are reported annually, and outbreaks occur sporadically^20^.

### Study population

This study involved febrile individuals who presented at selected hospitals and tested positive for malaria during the study period. As this was a descriptive and exploratory study, sample size and power calculations were precluded. However, the sample size was estimated according to Lwanga and Lemeshow^70^ based on the use of the following: a 95% confidence level, 5% significance level and a very high prevalence of malaria. Consequently, it was determined that 138 *P. falciparum* isolates were the required sample size for the objectives of this study. During the study period, a total of 530 febrile individuals agreed to take part in the study and were tested for malaria. Of them, 250 were found to be positive for *P. falciparum* parasites and were considered in the study. However, 227 isolates were successfully amplified for the *pfmdr1* gene and these were included in the final data analysis.

### Collection and examination of blood samples

About 2–3 ml of venous blood was collected from each participant into an EDTA tube that was labelled with the participant’s information and unique reference number. Shortly thereafter, thick and thin blood smears were prepared on a glass slide and stained with diluted Giemsa stain following a standard protocol^71^. Both smears were examined under a light microscope in order to detect and identify *Plasmodium* parasite species. The parasite density or parasitaemia level was calculated from the thick blood smear by counting the asexual stages of the malaria parasites in the sample against a white blood cell count of 200^71^. In addition, dried blood spots were prepared on appropriate filter papers (3MM Whatman International Ltd, England) and stored in labelled, separated, zipped plastic bags for later use in the molecular examination.

### Molecular analysis

Genomic DNA was extracted from the dried blood spots using a Qiagen blood and tissue kit (QIAGEN, DNeasy® Blood & Tissue Kit, Cat. no. 69506, Germany), according to the manufacturer’s instructions. DNA was eluted using 100 µl AE elution buffer (10 mM Tris–Cl; 0.5 mM EDTA; pH 9.0) provided in the kit and kept at − 20 °C until used.

### Detection of *pfmdr1* mutations

The genomic DNA of *P. falciparum* was amplified by PCR for the *pfmdr1* gene of antimalarial drugs resistance and subjected to restriction fragment length polymorphism (RFLP) for the detection of point mutations at codons 86 and 1246 of the *Pfmdr1* gene, according to an established protocol^72^. Moreover, mutations at codons 184, 1034, and 1042 of the *Pfmdr1* gene were analysed using a previously designed PCR–RFLP protocol^39^, with suitable modifications for codon 184 in the PCR amplification and RE digestion. A forward oligonucleotide primer MDR184-F (5′-GATAATAATCCTGGATCTAAATTAAGA-3′) was designed and utilized as an alternative of A4 to amplify a 155 bp fragment instead of 560 bp, and the use of Swa1 restriction enzyme as an alternative of Dra1, which cuts the amplicon once at the restriction site ATTT/AAA into 123 bp and 32 bp for the mutant alleles but not for the wild type alleles^36^.

Table 4 displayed information about primers sequences, PCR thermal conditions, and RE used for the detection of point mutations of the *pfmdr1* gene.

**Table 4:** Nested PCR–RFLP protocol for the detection of *Pfmdr1* point mutations.

Codon	PCR	Primer	Nucleotide sequence	Amplicon size	Thermal cycling conditions	Restriction enzyme	Target allele	Cleaves size in bp
86	Nest 1	MDR-A	GCGCGCGTTGAACAAAAAGAGTACCGCTG	450	94 °C /5 min; 25 cycles (94 °C/30 s, 50 °C/90 s, 65 °C/90 s) 65 °C/10 min	–		
		MDR-B	GGGCCCTCGTACCAATTCCTGAACTCAC					
	Nest 2	MDR-D1	TTTACCGTTTAAATGTTTACCTGC	291		Afl-III	Mutant	126 + 165
		MDR-D2	CCATCTTGATAAAAAACACTTCTT					
184	Nest 1	A1	TGTTGAAAGATGGGTAAAGAGCAGAAAGAG	657	94 °C /5 min 25 cycles (94 °C/30 s, 45 °C/60 s, 72 °C/60 s) 72 °C/5 min	–		
		A3	TACTTTCTTATTACATATGACACCACAAACA					
	Nest 2	A2	GTCAAACGTGCATTTTTTATTAATGACCATTTA	155		SwaI	Mutant	123 + 32
		MDR184-F	GATAATAATCCTGGATCTAAATTAAGA					
1034 & 1042	Nest 1	O1	AGAAGATTATTTCTGTAATTTGATACAAAAAGC	877	94 °C /5 min 25 cycles (94 °C/30 s, 45 °C/60 s, 72 °C/60 s 72 °C/5 min	–		
		O2	ATGATTCGATAAATTCATCTATAGCAGCAA					
	Nest 2	1034-F	AGAATTATTGTAAATGCAGCTTTATGGGGACTC	233		DdeI	Wild	Wild: 2 sites cut114 + 56 Mutant:1site 172 + 59
		1042-R	AATGGATAATATTTCTCAAATGATAACTTAGCA			AseI	Wild	
1246	Nest 1	1246-A	GGGGGATGACAAATTTTCAAGATTA	295	94 °C /5 min 25 cycles (94 °C/30 s, 50 °C/90 s, 65 °C/90 s) 65 °C/10 min		–	
		1246-B	GGGGGACTAACACGTTTAACATCTT					
	Nest 2	1246-D1	AATGTAAATGAATTTTCAAACC	202		Bg1 II	Wild	111 + 90
		1246-D2	CATCTTCTCTTCCAAATTTGATA					

Genomic DNA of *P. falciparum* reference strains provided by Malaria Research and Reference Reagents Resources Centre (MR4, ATCCW, Manassas VA, USA) were utilized as positive controls. The genomic DNA of *P. falciparum* strain Dd2 (MRA-150G) and HB3 (MRA-155G) were employed as positive controls for the 86 and 184 and 1042 mutant alleles, respectively. Likewise, the *P. falciparum* reference strains 3D7 (MRA-102G) was employed as positive control for the wild types of *pfmdr1* at all codons.

### Statistical analysis

Data were entered into Microsoft Office Excel 365 spreadsheets and then imported into IBM SPSS v20 (IBM Corp., NY, USA) for statistical analysis. Categorical variables including the point *pfmdr1* mutations and haplotypes (dependent variables) and the patients’ demographic factors, were expressed as frequencies and percentages. Quantitative variables including age (years) and parasitaemia levels were expressed as mean ± standard deviation (SD) or median (interquartile range, IQR). Pearson’s Chi-square (*χ*^2^) test or Fisher’s exact test were used, where applicable, to examine the associations between the dependent and the independent variables. A *P-*value of < 0.05 was set as the statistically significant cut-off value for all tests.

## Data Availability

The data that support the findings of this study are available from the corresponding author upon reasonable request.
